# The Mechanism of *Dendrobium officinale* as a Treatment for Hyperlipidemia Based on Network Pharmacology and Experimental Validation

**DOI:** 10.1155/2022/5821829

**Published:** 2022-04-13

**Authors:** Lin-Zi Li, Hui-Ying Wang, Jia-Hui Huang, Kun Liu, Xiao-Jie Feng, Xi-Ming Wang, Li-Jie Zhu, Xing-Lishang He, Xiang Zheng, Hai-Long Li, Ying-Jie Dong, Bo Li, Han-Song Wu, Ning-Hua Jiang, Gui-Yuan Lv, Su-Hong Chen

**Affiliations:** ^1^Zhejiang University of Technology, Hangzhou, Zhejiang 310014, China; ^2^The Second Affiliated Hospital of Jiaxing University, Huancheng North Road, Nanhu District, Jiaxing, Zhejiang 314001, China; ^3^Zhejiang Chinese Medical University, Hangzhou, Zhejiang 310053, China

## Abstract

**Materials and Methods:**

The active compounds in DO, their targets, and targets associated with hyperlipidemia were screened across various databases, and the hidden targets of DO in treating hyperlipidemia were forecast. The compound-target (C-T), protein-protein interaction (PPI), and compound-target-pathway (C-T-P) networks of DO were set up with Cytoscape software. The hub genes and core clusters of DO predicted to be active against hyperlipidemia were calculated by Cytoscape. The DAVID database was adopted for Gene Ontology (GO) analysis and KEGG pathway enrichment analysis. Next, we used the high-sucrose-fat diet and alcohol (HFDA)-induced hyperlipidemia rats to evaluate the hypolipidemic effect of DO.

**Results:**

In this study, we obtained 264 compounds from DO, revealed 11 bioactive compounds, and predicted 89 potential targets of DO. The network analysis uncovered that naringenin, isorhamnetin, and taxifolin might be the compounds in DO that are mainly in charge of its roles in hyperlipidemia and might play a role by modulating the targets (including PPARG, ADIPOQ, AKT1, TNF, and APOB). The pathway analysis showed that DO might affect diverse signaling pathways related to the pathogenesis of hyperlipidemia, including PPAR signaling pathway, insulin resistance, AMPK signaling pathway, and non-alcoholic fatty liver disease simultaneously. Meanwhile, in the HFDA-induced hyperlipidemia rat model, DO could significantly decrease the level of TC, TG, LDL-c, and ALT in serum, and increase HDL-c as well. The liver pathological section indicated that DO could ease liver damage and lipid cumulation.

**Conclusion:**

In summary, the biological targets of the main bioactive compounds in DO were found to distribute across multiple metabolic pathways. These findings suggest that a mutual regulatory system consisting of multiple components, targets, and pathways is a likely mechanism through which DO may improve hyperlipidemia. Validation experiments indicated that DO may treat hyperlipidemia by affecting NAFLD-related signaling pathways.

## 1. Introduction

As an abnormality of lipid metabolism, hyperlipidemia has characteristics such as an increase of total cholesterol (TC), triglyceride (TG), and low-density lipoprotein cholesterol (LDL-c), and/or a decrease in high-density lipoprotein cholesterol (HDL-c) in circulating plasma. Hyperlipidemia is associated with etiopathogenesis of different diseases, including atherosclerosis, hypertension, metabolic syndrome, and cardiovascular disease (CVD) [1]. Numerous studies have demonstrated that dyslipidemia characterized by elevated LDL-c or TC presents a high risk of CVD [2]. Indeed, lowering LDL-c levels can significantly reduce the risk of arteriosclerotic cardiovascular disease (ASCVD) morbidity and mortality [3]. Other types of dyslipidemia, such as elevated TG or decreased HDL-c, are also associated with increased ASCVD risk [4]. Therefore, the effective control of dyslipidemia is important for the prevention of cardiovascular and cerebrovascular diseases.

Monotherapy with pharmacologic agents is inherently challenging for the treatment of hyperlipidemia. Indeed, hyperlipidemia requires a comprehensive approach including changes in diet, exercise, and pharmaceutical therapy. Currently, the commonly used lipid-lowering drugs such as statins and fibrates usually have side effects and contraindications with long-time application, and many clinicians would prefer their patients to regulate dyslipidemia through healthy lifestyle modifications [5]. In contrast to the potential toxicity posed by pharmacologic agents, the alternative treatments and traditional herbal medicines used to treat hyperlipidemia are exceptionally safe [6]. In China and many East Asian countries, traditional Chinese medicine (TCM) is extensively adopted for the prophylaxis and treatment of hyperlipidemia in clinical practice [7]. Indeed, TCM is thought to present several unique advantages for prevention or treatment of hyperlipidemia, due to its action through multiple components, approaches, and targets [8].

*Dendrobium officinale* (DO) is the most valuable species in the Dendrobium genus, which is mainly observed in the Zhejiang, Yun-nan, and Guangxi Province of China [9]. Its stem has been conventionally used as both food and medicine for centuries of clinical use, and it is also noted in the “Pharmacopoeia of the People's Republic of China” [10]. Recent pharmacological studies have suggested that it may have a hepatoprotective effect, enhance immunity, anti-oxidant properties, and hypoglycemic effect, and protect against gastric ulcers [11–15]. Remarkably, increasing evidence has shown that DO can also modulate the blood-lipid disorder in ApoE^−/−^mice [16] and in other hyperlipidemic models [17]. Our previous studies [11, 18] revealed that DO reduces serum aspartate aminotransferase (AST) and alanine aminotransferase (ALT). Those outcomes indicate that DO has strong potential as a therapeutic for hyperlipidemia. However, the material basis and mechanism of action remain unclear and require further study.

Network pharmacology is a new approach of “active compound-target- disease” interaction network based on multidisciplinary integration theory [19]. Complicated diseases such as metabolic diseases are not due to single mutations or dysfunction of a single signaling pathway. Instead, they are brought by multiple mutations or dysfunction of an entire regulatory network. Unlike the “one drug, one target” approaches, network pharmacology pays attention to the reality that a lot of active ingredients may interact with various diverse genes or proteins, this analytical principle shared with the holistic approach of TCM [20]. Network pharmacology can predict the effects of drugs on potential molecular disease networks in systematical and comprehensive way, enabling observation of the of multi-component, multi-pathway, and multi-target synergies that may be afforded by TCM [21].

Therefore, the molecular mechanism of DO as a treatment for hyperlipidemia was clarified with network pharmacology. Furthermore, we validated experiments in hyperlipidemic rats induced with high-sucrose-fat diet and alcohol (HFDA) to further verify the effect of DO in ameliorating hyperlipidemia. These findings may lay a foundation for further exploration of the therapeutic value of DO as a treatment for hyperlipidemia and other clinical applications.

## 2. Materials and Methods

**Figure 1:**
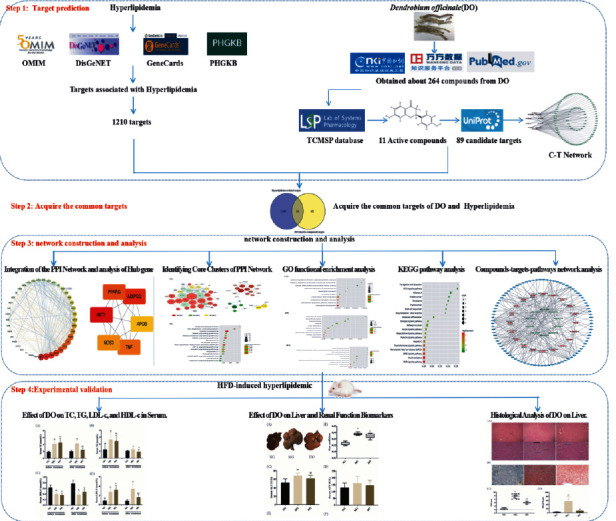
Workflow of Systems Pharmacology Research of DO on hyperlipidemia. The active compounds of DO were conducted with various bibliographical databases, and their potential targets were recognized by TCMSP database. Next, the genes associated with hyperlipidemia were filtered by the OMIM, DisGeNET, GeneCards, and PHGKB database. The “compound-target,” protein-protein interaction (PPI), and “compound-target-pathway” network of DO was constructed by Cytoscape software. The hub genes and core clusters of DO against hyperlipidemia were calculated by Cytoscape software. DAVID database was adopted for Gene Ontology (GO) and KEGG pathway enrichment analyses.

This research adopted network pharmacology and validation experiments to unveil the biochemistry basis and underlie mechanisms of DO as a treatment for hyperlipidemia.  Figure 1 shows an overview of the experimental steps.

### 2.1. Network Pharmacology Analysis

#### 2.1.1. Data Preparation and Active Compounds Screening

A comprehensive search of DO's chemical compounds was set up with different bibliographical databases such as China National Knowledge Internet (CNKI: http://www.cnki.net), Wanfang Database (http://www.wanfangdata.com.cn/index.html), and PubMed (http://www.ncbi.nlm.nih.gov). Then, analyzing and collating the literature to obtain the chemical constituents of DO were performed.

All DO's compounds were input into PubChem (https://pubchem.ncbi.nlm.nih.gov/) [22] and Traditional Chinese Medicine Systems Pharmacology Database and Analysis Platform (TCMSP, http://tcmspw.com/index.php) [23] to get the 3D molecular structure files. Because we failed to predict the targets of the compounds without accurate structural data successfully, these chemical data without precise structural information are deleted.

We used the TCMSP to analyze the DO compounds collected above, and two silico ADME models were used to obtain the active ingredients in DO, including Per OB (forecast of oral bioavailability) and Per DL (forecast of drug-likeness). The thresholds of the two screening models were set as Per OB ≥ 30% and Per DL ≥ 0.18. The obtained active compounds were adopted as the candidate active compounds [24].

#### 2.1.2. Targets for the Active Compounds

TCMSP was adopted to screen the targets of candidate active substances in DO. The collected targets were confirmed with the Uniprot protein sequence resource (http://www.Uniprot.org/), including name, gene ID, and organism. The active compounds without targets were eliminated; the active compound-target dataset was set up.

#### 2.1.3. Construction of Active Compound-Target Network

Cytoscape 3.7.0 platform (http://cytoscape.org/) [25] provided the candidate active compounds and their potential targets to set up the active compound-target network. The key architecture of the Cytoscape 3.7.0 platform is a network with a gene, protein, or molecule as each node, and the associations between the nodes refer to the mutual effect between these biological explorations [26].

#### 2.1.4. Predicting the Targets of Hyperlipidemia

In this study, the disease targets of hyperlipidemia were obtained by searching four databases, including GeneCards database (https://www.genecards.org/) [27], OMIM database (https://omim.org/) [28], DisGeNET database (http://www.disgenet.org/) [29], and PHGKB database (https://phgkb.cdc.gov/PHGKB/startPagePhenoPedia.action/) [30].

Disease targets were collected using “hyperlipidemia” or “HLP” as keywords. In GeneCards database, target genes were chosen with the Relevance Score ≥1, and in DisGeNET database, target genes were chosen with the Gene-Disease Score ≥0.1. After duplicates were deleted, hyperlipidemia-related targets were obtained [31].

#### 2.1.5. Protein-Protein Interaction (PPI) Network Construction and Hub Gene Analysis

The Venn diagram was set up through an online website (https://bioinfogp.cnb.csic.es/tools/venny/index.html) to acquire the common targets for DO bioactive compound targets and the hyperlipidemia associated targets.

Then, the common target genes were input into the STRING database (http://string-db.org/) [32] to explore the protein interaction, and the PPI network was constructed with the Cytoscape. The nodes in PPI network were performed by “Network Analyzer,” and the Hub genes of DO against hyperlipidemia were calculated by CytoHubba (http://apps.cytoscape.org/apps/cytohubba) [33] plugin by MCC algorithm in this PPI network.

#### 2.1.6. Identifying Core Clusters of PPI Network

In the complicated biological data network, several genes or proteins are closely associated with each other with the same or similar functions, so they can exert a significant biological coordination effect as a cluster. The data of every node in the network assists in analyzing clusters and constructing functional modules [34]. The functional modules of the PPI network in the hyperlipidemia treated with DO were selected by using the Molecular Complex Detection (MCODE) (a plugin in Cytoscape, https://apps.cytoscape.org/apps/mcode).

#### 2.1.7. Gene Ontology (GO) and Kyoto Encyclopedia of Genes and Genomes (KEGG) Enrichment Analysis

As an effective bioinformatics tool, the GO analysis can characterize molecular function (MF), cellular components (CC), and biological process (BP) of genes [35]. The KEGG enrichment exploration collects databases illustrating biological paths, genomes, drugs, and diseases [36]. The Database for Annotation, Visualization and Integrated Discovery (DAVID, https://david.ncifcrf.gov/), which is an integrated functional annotation tool, was used to know the biological significance behind the large gene datasets [37]. In this study, the GO function and KEGG pathway enhancement of proteins taking part in PPI network was analyzed with DAVID database.

#### 2.1.8. Compound-Target-Pathway for DO as a Treatment for Hyperlipidemia Network Construction

We used Cytoscape software to construct the “Compounds-targets-pathways (C-T-P)” network for DO as a treatment for hyperlipidemia according to the active compounds in DO, intersecting the targeted genes related hyperlipidemia with the DO active compounds through the pathway from KEGG analysis. In this network, every compound, target, or pathway is represented by a node, and every interaction by an edge. At the same time, the plugin Network Analyzer of Cytoscape 3.7.0 was adopted to analyze the degree representing the number of edges interacting with the node.

### 2.2. Experimental Validation

#### 2.2.1. Chemicals and Reagents

Biochemical reagents as TC, TG, HDL-c, ALT, and AST were purchased from Meikang Biotechnology Co. (Ningbo, Zhejiang, China). Nanjing technology Co., Ltd. (Jiangsu, China) offered hematoxylin and eosin (HE).

Zhejiang Senyu Co., Ltd. (Zhejiang China) offered DO, the origin is Zhejiang (Yiwu, Zhejiang), and the growing age is three years old. DO was pulverized into powder (D_90_ size distribution of 35.01 ± 1.19 *μ*m) and pure water used to prepare with a concentration of 60 mg/mL (crude drug) for reserve. High-sucrose-fat diet (lard 10%, cholesterol 2%, bile salt 0.5%, egg yolk powder 5%, sucrose 10% and basic diet 72.5%; *w/w*) and basic diet were all produced by Zhejiang Academy of Medical Science (Hangzhou, China).

#### 2.2.2. Animals and Experimental Design

Animal Supply Center of Zhejiang Academy of Medical Science (20200907Aazz0100018868, Hangzhou, China) offered Sprague-Dawley (SD) rats (*n* = 30). The hyperlipidemia model of rats induced by high-sucrose-fat diet and alcohol (HFDA) and the specific methods were as follows: the rats were fed with high-sugar and high-fat diet (normal diet 76.5%, fructose 10%, edible lard 10%, cholesterol 1.2%, bile salt 0.25%); at the same time, Red Star (Hongxing) and Erguotou (alcohol volume fraction gradually increased from 4% to 22%) were added into the drinking water of the rats; 4% alcohol was given for 4 consecutive days at the beginning of modeling, grew to 8% on the fifth day, and then increased by 4% every other 3 days until 22% (Table 1). Once the extents of TC, TG, and LDL-C in serum greatly grew, and the differences were significant compared with the normal group (NG), indicating that the hyperlipidemia model was successfully constructed, all animals are raised under standard environmental conditions and comply with the Rules for the Use and Care of Laboratory Animals.

Then, SD rats were firstly fallen into 3 groups (*n* = 10): (1) normal control group (NG); (2) hyperlipemia model group (MG); (3) DO treatment group (DO, 600 mg/kg/d, P.O.). The NG rats received the basic diet and water throughout the whole experiment, and the remaining twenty rats were supplied with HFDA. After modeling for 8 weeks, the HFDA-induced hyperlipidemic rats were randomly assigned to 2 groups (MG and DO) according to the serum TC level, and then the MG and DO groups continued to be supplied with HFDA. NG and MG were given corresponding distilled water by intragastric administration, and DO was given DO (600 mg/kg/d, P.O.). During the experiment, the record of body weight was made every week. After 6 weeks of administration, the blood was collected through orbital vein after 12 hours of fasting. After the experiment, the livers were weighed, and the biggest lobes of livers were fixed with 4% paraformaldehyde, and the rest were kept at −80°C until the next use.

#### 2.2.3. Determination of TC, TG, LDL-c, HDL-c, AST, and ALT in Serum

The serum was centrifuged at 3500 rpm for 10 min at 4°C. The levels of TC, TG, HDL-C, ALT, and AST in serum were detected by automatic biochemical analyzer (Hitachi 7020, Japan). Friedewald's formula as LDL-c = TC - (HDL-c + TG/2.2) [38] was adopted to calculate serum LDL-c.

#### 2.2.4. Histological Analysis

Liver histopathology was evaluated with hematoxylin-eosin staining (H&E) and Oil red O staining. The liver specimens were fixed in 4% paraformaldehyde, embedded in paraffin, and sliced at 3 µm thickness, and then, the staining of sections acquired was made with hematoxylin and eosin (H&E) for the histological test [39]. 0.2% Oil-Red O was employed to stain cryosections of liver, followed by counterstaining with hematoxylin for visualizing the lipid droplets. Biological microscope (Olympus BX43, Japan) was adopted to observe tissue sections, followed by analysis by Image-Pro Plus software.

## 3. Statistical Analysis

All measurements in this research were shown as means ± standard deviation and subjected to one-way analysis of variance (ANOVA). *P* < 0.05 was of statistical significance. SPSS 17.0 statistical software was adopted to perform all analyses.

## 4. Results

### 4.1. Screening for Active Compounds in DO

According to comprehensive search by using various bibliographical databases, we obtained about 264 compounds in DO; the main chemical compounds in DO are phenanthrenes, bibenzyls, phenols, acids, esters, amides, saccharides, glycosides, essential oils, and so on (for more detailed information, see Supplementary Table S1: chemical compounds in DO).

We input 264 compounds of DO into PubChem and TCMSP to get the 3D molecular structure files and removed these chemical data that were without precise chemical structural information. Eventually, 139 compounds with chemical structural information were reserved for further study (Supplementary Table S2: 139 compounds with structural information in DO). Two silico ADME models (Per OB ≥ 30% and Per DL ≥ 0.18) were used to analyze the DO compounds collected above, and we have got 11 active compounds finally (Table 2).

### 4.2. Compound-Target (C-T) Network Analysis

The active compounds with no targets on basis of the TCMSP database or that had no related gene name on basis of the Uniprot database were removed. In the end, 10 compounds and 89 candidate targets were obtained according to Table 3.

**Figure 2:**
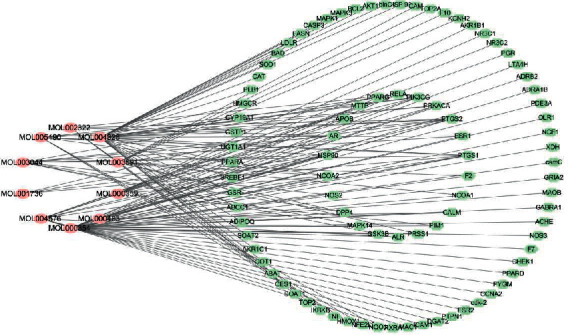
Compound-Target network. There were 99 nodes (10 bioactive compound nodes, 89 target nodes) and 141 edges in this network, and the red node refers to the compounds, and the green stands for the targets. Mean extent of per compound was 7.8, aringenin (MOL004328, degree = 37) and isorhamnetin (MOL000354, degree = 37) have a higher degree, showing more mutual effects with targets and might be the core active compounds on anti-hyperlipidemia. Besides, ingredients were actived by correlating candidate targets of PTGS2 (degree = 9), PTGS1 (degree = 7), HSP90 (degree = 7), PIK3CG (degree = 5) and PRKACA (degree = 4).

A visualized Compound-Target network (C-T network) diagram was established on basis of hidden ingredients and targets by Cytoscape software, and it is shown in Figure 2. There were 99 nodes (10 bioactive compound nodes, 89 target nodes) and 141 edges in this network. The mean extent of per compound was 7.8, and naringenin (MOL004328, degree = 37) and isorhamnetin (MOL000354, degree = 37) have a higher extent, showing more mutual effects with targets, and might be the core active compounds on anti-hyperlipidemia.

### 4.3. Potential Targets of Hyperlipidemia

Through these four databases, GeneCards, DisGeNET, OMIM, and PHGKB, we obtained 802, 69, 102, and 545 hyperlipidemia-associated targets, respectively. Concluding the outcomes of different databases and eliminating duplicate genes, 1210 targets associated with hyperlipidemia were picked up. The details of hyperlipidemia-associated targets were offered in Supplementary (Supplementary Table S3: the detailed information of hyperlipidemia-related targets).

### 4.4. Integration of the PPI Network and Analysis of Hub Gene

**Figure 3:**
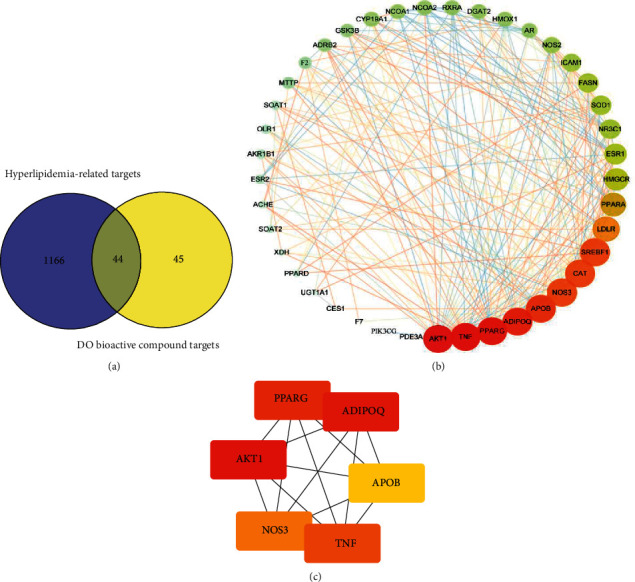
Venn diagram of targets and PPI network of DO treating hyperlipidemia. (A) A Venn diagram was set up by an online website (https://bioinfogp.cnb.csic.es/tools/venny/index.html) to acquire the 44 common targets of the DO bioactive component targets and the hyperlipidemia-associated targets. (B) These target genes were inputted into the STRING online website (PPI score > 0.4), and the PPI network made up of 44 inter-action nodes and 245 interaction edges. Nodes refer to core target genes. The size of the nodes and edges matches the value of degree and integrate mark respectively. The color of the nodes refers to the value of degree. If the color become darker (red), the degree will be higher. (C) Hub gene of DO against hyperlipidemia was calculated by Cytohubba (http://apps.cytoscape.org/apps/cytohubba ) plugin by MCC algorithm, the five nodes with the largest degree value were chosen as the hub genes, the darker (red) the node color, the higher the score, that were, RAC-alpha serine/threonine-protein kinase (AKT1), Tumor necrosis factor (TNF), Peroxisome proliferator activated receptor gamma (PPARG), Adiponectin (ADIPOQ), Apolipoprotein B-100 (APOB) and Nitric-oxide synthase endothelial (NOS3).

The Venn diagram was set up by an online website (https://bioinfogp.cnb.csic.es/tools/venny/index.html) to acquire the 44 common targets for DO bioactive compound targets and the hyperlipidemia related targets (Figure 3(a)).

We input these common genes into the STRING online website (PPI score >0.4) to create the PPI network, and finally the network made up of 44 interaction nodes and 245 interaction edges were created. As shown in Figure 3(b), the size of the nodes and edges correspond to the value of degree and integrate mark. The color of the nodes indicates the value of degree. In case of darker color (red), higher degree was indicated.

The Hub genes were screened by CytoHubba (the plug-in based on Cytoscape) from the interaction network. And then, we used MCC algorithm to find out the top 6 Hub genes of DO as a treatment for hyperlipidemia (Figure 3(c)), which were RAC-alpha serine/threonine-protein kinase (AKT1), Tumor necrosis factor (TNF), Peroxisome proliferator activated receptor gamma (PPARG), Adiponectin (ADIPOQ), Apolipoprotein B-100 (APOB), and Nitric-oxide synthase endothelial (NOS3). These hub genes were input into the DisGeNET database to acquire the protein class of genes according to Table 4.

### 4.5. Identification of Core PPI Clusters

**Figure 4:**
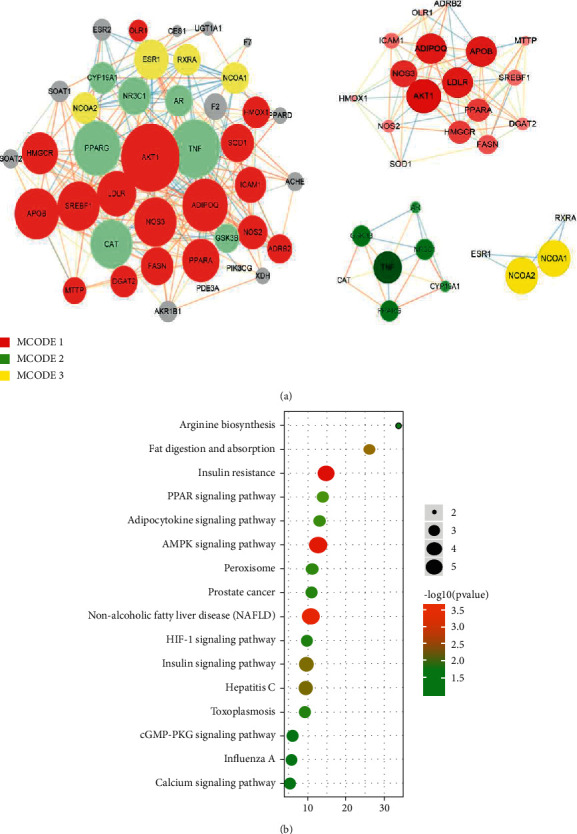
GO Enrichment Analysis with DAVID database. (a) Key network clustering diagram of DO for hyperlipidemia; (b) KEGG enrichment analysis was performed on core targets. On basis of the MCODE clustering analysis, the key PPI network of musk for ischemic stroke could be fallen into 3 modules. According to the Figure 4(a), the red node represents the MCODE 1, the green node represents the MCODE 2 and the yellow node represents the MCODE 3. KEGG enrichment analysis of cluster 1 (MCODE 1) was made. Top 10 KEGG enrichment pathways were framed in a bubble plot on basis of the *P* value (Figure 4(b)).

On basis of the MCODE clustering exploration, the key PPI network of DO for hyperlipidemia could be fallen into 3 modules (Figure 4(a)). There were 21 genes in cluster 1 (MCODE 1), mark 7.17, and the key gene was Apolipoprotein B-100 (APOB); there were 7 genes in cluster 2 (MCODE 2), mark 5.80, and the key gene was Glucocorticoid receptor (NR3C1); there were 4 genes in cluster 3 (MCODE 3), mark 4.33, and the key gene was Retinoic acid receptor RXR-alpha (RXRA).

KEGG enrichment exploration was conducted on cluster 1 (McOde1). According to the *P* value, top 10 KEGG enrichment pathways were acquired and framed in a bubble plot (Figure 4(b)). As shown in figure, those genes of cluster 1 were related with the insulin signaling pathway, AMPK signaling pathway, and nonalcoholic fatty liver disease (NAFLD).

### 4.6. GO Functional Enrichment and KEGG Pathway Analysis

**Figure 5:**
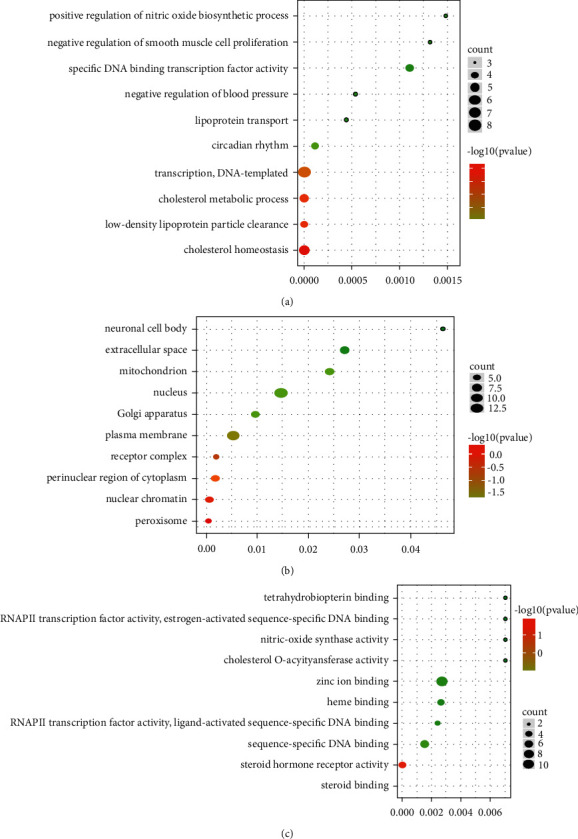
GO functional enrichment analysis with DAVID database. (a) The biological processes enrichment analysis, (b) The molecular functions enrichment analysis, (c) The cellular component enrichment analysis. Intersection targeted genes associated with hyperlipidemia and the DO active compounds associated as bits were adopted to fish corresponding functions from DAVID, import target genes into DAVID database for GO analysis biological process. *Y*-axis stood for greatly enhanced biological process categories associated with target genes, and *X*-axis referred to the log10 (*P* value), The size of the dot means the number of target genes in the pathway, and the color of the dot stands for various FDR scopes.

To elucidate the multiple mechanisms of DO in treating hyperlipidemia from an integrated level, GO enrichment analysis was made on the biological process, molecular function, and cellular component of the 44 common targets.  Figure 5 showed the top 10 greatly enhanced GO terms of these targets (FDR < 0.05). The outcomes showed that the targets of DO were greatly related to 5 biological processes (BP): cholesterol homeostasis, low-density lipoprotein particle clearance, cholesterol metabolic process, and transcription DNA-templated and circadian rhythm; 5 molecular functions (MF): peroxisome, nuclear chromatin, perinuclear region of cytoplasm, receptor complex, and plasma membrane; 5 cellular components (CC): steroid binding, steroid hormone receptor activity, sequence-specific DNA binding, heme binding, and zinc ion binding.

**Figure 6:**
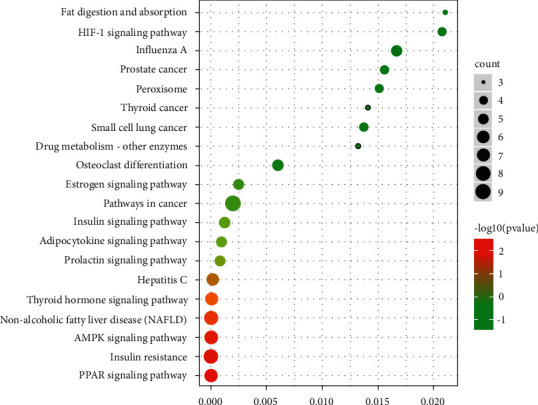
KEGG pathway analysis with DAVID database. (a) The GO enrichment analysis; (b) KEGG pathway enrichment analysis. Intersection targeted genes associated with hyperlipidemia and the DO active compounds associated as bits were adopted to fish corresponding functions from DAVID, import target genes into DAVID database for KEGG pathway analysis. *Y*-axis referred to greatly improved biological process categories associated with target genes, and *X*-axis stood for the log10 (*P* value), The size of the dot means the number of target genes in the pathway, and the color of the dot shows the different FDR scope.

As shown in Figure 6, we analyzed the top 20 significantly enriched KEGG pathways of these targets (FDR < 0.05). The result indicated that the targets are mostly associated with signal pathways including PPAR signaling pathway, insulin resistance, AMPK signaling pathway, nonalcoholic fatty liver disease (NAFLD), and thyroid hormone signaling pathway.

### 4.7. Compound-Target-Pathway for DO against Hyperlipidemia Network Analysis

**Figure 7:**
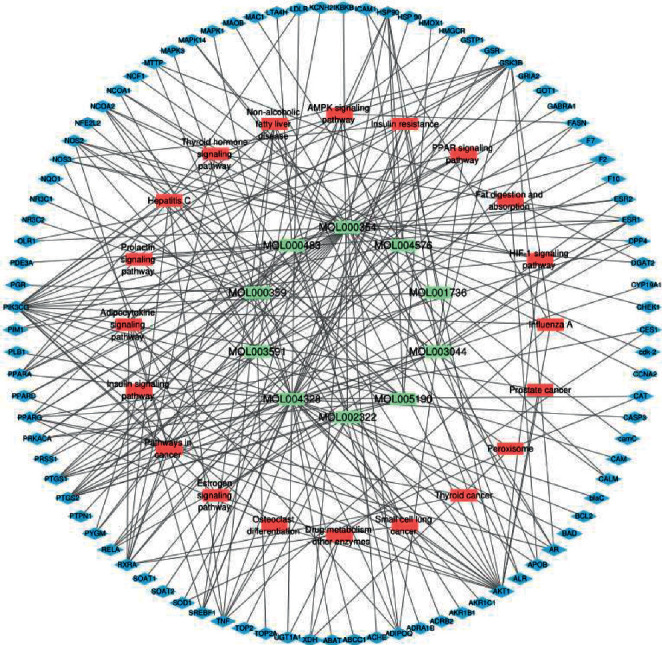
Compounds-targets-pathways for DO against hyperlipidemia network analysis. By assembling the core pathways acquired by analyzing C-T-P network (the green node refers to the compound, the red stands for the pathway, and the blue means the target), naringenin (MOL004328, degree = 37), isorhamnetin (MOL000354, degree = 37) and taxifolin (MOL004576, degree = 12) have a higher degree, indicating more mutual effect with targets and signaling pathway might be the core active compounds on anti-hyperlipidemia. By analyzing C-T-P network, we picked out 5 important signaling pathways that were significantly associated with DO treatment of hyperlipidemia. The 5 of chosen pathways including insulin resistance (degree = 8), Nonalcoholic fatty liver disease (degree = 8), Pathways in cancer (degree = 8), AMPK signaling pathway (degree = 7) and thyroid hormone signaling pathway (degree = 7).

To construct the “Compound-target-pathway (C-T-P)” network as shown in Figure 7, we assembled the key pathways by analyzing C-T-P network. Naringenin (MOL004328, degree = 37), isorhamnetin (MOL000354, degree = 37), and taxifolin (MOL004576, degree = 12) possess higher degrees, showing that more mutual effects with targets and signaling pathways might be the core active compounds on anti-hyperlipidemia.

By analyzing C-T-P network, we picked out 5 important signaling pathways that were significantly associated with DO as a treatment for hyperlipidemia. As shown in Table 5, the 5 chosen pathways included insulin resistance (degree = 8), nonalcoholic fatty liver disease (degree = 8), pathways in cancer (degree = 8), AMPK signaling pathway (degree = 7), and thyroid hormone signaling pathway (degree = 7).

### 4.8. Effect of DO on TC, TG, LDL-c, and HDL-c in Serum

**Figure 8:**
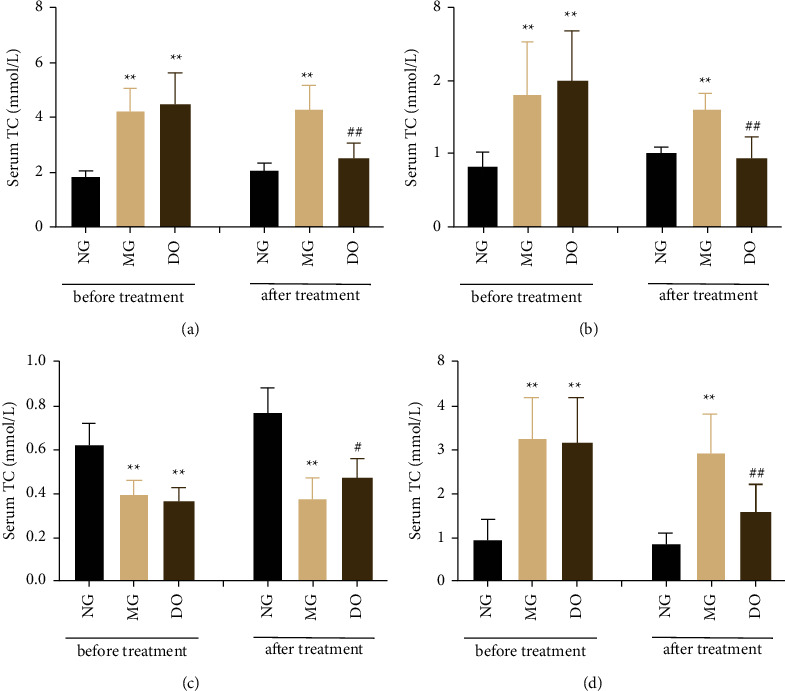
Role of DO in TC, TG, LDL-c, and HDL-c in Serum. (a) Roles of DO in serum TC before and after treatment. (b) Effects of DO on serum TG before and after treatment. (c) Effects of DO on serum HDL-c before and after treatment. (d) Effects of DO on serum LDL-c before and after treatment. Values are shown as mean ± SD. ^#^*P* < 0.05 vs. MG and ^*∗*^*P* < 0.05 vs. NG.

To examine the effect of DO on the blood lipid degree in HFDA-induced hyperlipidemia rats, serum TC, TG, LDL-c, and HDL-c both were measured before and after DO administration. As shown in Figure 8, after modeling for 8 weeks, by comparing with NG before treatment, the serum TG, TC, and LDL-c all greatly grew, and the serum HDL-c greatly decreased in HFDA-induced hyperlipidemia rats (*P* < 0.01). After administrating the DO for 6 weeks, the serum TC, TG, and LDL-c significantly decreased, and the serum HDL-c was elevated in a significantly different way (*P* < 0.05 − 0.01) by comparing with the MG. These results indicate that DO can decrease the serum TC, TG, and LDL-c and increase the serum HDL-c to influence the blood lipid.

### 4.9. Effect of DO on Liver Function Biomarkers

**Figure 9:**
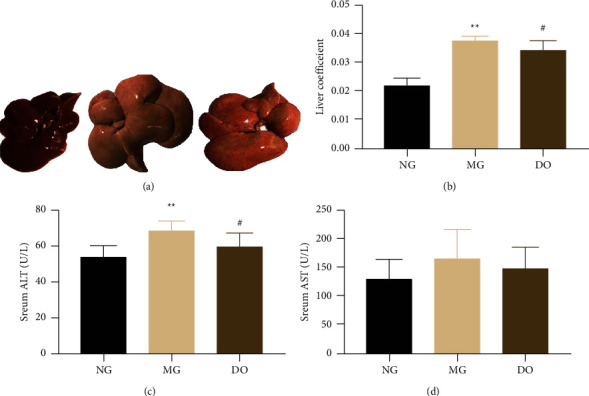
Role of DO in Liver and Renal Function Biomarkers. (a) Liver photographs. (b) Liver index. (c) Effects of DO on serum ALT after treatment. (d) Effects of DO on serum AST after treatment. Values are shown as mean ± SD. ^#^*P* < 0.05 vs. MG and ^*∗*^*P* < 0.05 vs. NG.

Long-term disturbed lipid homeostasis can lead to hepatic lipid lesions. We detected serum AST and ALT levels after administration of DO to decide the liver function.  Figure 9 displays that, by comparing with NG, the serum ALT (Figure 9(c)) grew greatly in MG rats (*P* < 0.01) without impact on AST (Figure 9(d)). After administration of DO for 6 weeks, the serum AST in DO group significantly decreases compared with MG (*P* < 0.05) (Figure 9(c)), but no impact on AST (Figure 9(d)) was shown. These outcomes suggest that DO can improve liver damage caused by lipid metabolism disorder to a certain extent.

Compared with NG, the liver weight was greatly grown in MG rats, and by comparing with MG, the liver index in treatment group was significantly decreased (Figure 9(b)). The liver of NG rats was reddish-brown, while the liver of MG rats was yellow with obvious white spots on its surface, which shows hepatic steatosis. On the contrary, the liver improved significantly in the DO group, as shown in Figure 9(a).

### 4.10. Histological Analysis of DO on Liver

**Figure 10:**
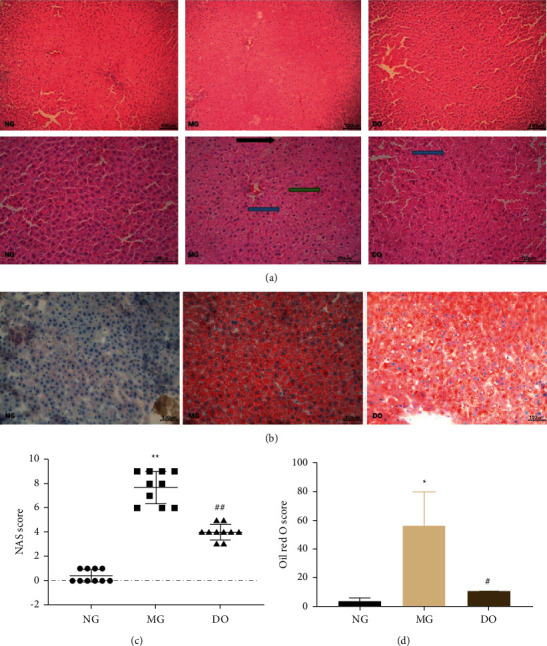
Histological Analysis of DO on Liver. (a) Representative photomicrographs of HE staining (×200 and ×400). The blue arrow refers to steatosis, the green arrow stands for lobular inflammation, the black arrow means ballooning degeneration. (b) Representative photomicrographs of Oil Red O staining (×200). (c) The NAS scores. (d) The OD of Oil Red O staining (fold change). Values are shown as mean ± SD. ^#^*P* < 0.05 vs. MG and ^*∗*^*P* < 0.05 vs. NG.

In this research, 10 photomicrographs of HE-staining were chosen from each group to calculate the NAS mark at a magnification of ×400. According to liver sections in NG, the structures of tissues were normal in polygonal edge, clear cell boundary, and clear round nucleus (Figure 10(a)). On the contrary, the MG showed visible histological variations such as cellular edema, focal degeneration, and necrosis. Similarly, despite degeneration, DO group is significantly better than the MG group. However, the tissues in DO have recovered to some extent, and cell edema was nearly observed with unique and clear tissue boundaries. Meanwhile, the NAS mark in the MG was greatly higher than the NG, while it declined after DO treatment; this showed that DO greatly attenuated inflammation, steatosis, and swelling of liver tissue caused by long-term dyslipidemia (Figure 10(c)).

Oil-red O staining showed the presence of lipid cumulation in both MG and DO groups compared with NG, but the lipid cumulation in DO group was less than that in MG group (Figures 10(b) and 10(d)).

## 5. Discussion

Hyperlipidemia is global threat to public health, contributing to significant annual mortality and enormous health care costs. DO is a tonic herb described in the Chinese Compendium of Materia Medica and has a longstanding history of use as a health food for the folk treatment of diseases related to yin-deficiency for decades, and modern pharmacological studies have confirmed that DO can lower blood lipids in animal models [16, 17].

Network pharmacology approach is a useful approach to research the bioactive compounds and mechanisms of TCM in treating hyperlipidemia. This approach combines insights about the drugs, target proteins, and diseases to form drug-target-disease networks, which are similar to the TCM principles of multi-component, multi-pathway, and multi-target synergy [40]. This research explored the hidden active compounds and useful mechanisms of DO in treating hyperlipidemia through network pharmacological exploration and experimental verification, aiming to provide theoretical evidence for developing DO as an adjuvant therapy for hyperlipidemia.

In this research, we obtained 11 bioactive compounds in DO from various bibliographical databases and predicted 89 potential targets, through the network (C-T, C-T-P, PPI) and KEGG pathway analyses, and specifically found that naringenin (MOL004328), isorhamnetin (MOL000354), and taxifolin (MOL004576) might be the main compounds in DO that are responsible for its effects on hyperlipidemia. These compounds may act by modulating the above targets (including AKT1, TNF, PPARG, ADIPOQ, and APOB). The pathway exploration in our study suggests that DO may effect multiple signaling pathways related to the pathogenesis of hyperlipidemia, including the PPAR signaling pathway, insulin resistance, AMPK signaling pathway, nonalcoholic fatty liver disease (NAFLD), and thyroid hormone signaling pathways simultaneously. The GO enrichment analysis of targets revealed that the ingredients of DO may have a synergistic effect on the treatment of hyperlipidemia, mainly by regulating cholesterol homeostasis, affecting cholesterol metabolic processes, and aiding in the clearance of low-density lipoprotein particles. Meanwhile, in the HFDA-induced hyperlipidemia rat model, DO could significantly decrease the level of TC, TG, LDL-c, and ALT, increase HDL-c in serum as well, and ease liver damage and lipid cumulation.

### 5.1. Active Compounds of DO against Hyperlipidemia

Several of the active compounds predicted to be active by the analyses in this study have actually been shown to be effective in reducing serum TC and lipid deposition.

For example, naringenin is a regulator for cholesterol efflux that enhances lipoprotein profiles and protects against cardiovascular disease, and that regulation is mediated by the ATF6 branch of the ER stress and PI3K/AKT pathway [41]. Importantly, recent studies support the role for naringenin in the treatment of dyslipidemia, hepatic steatosis, obesity, and atherosclerosis [42]. Recent research has indicated that isorhamnetin reduces serum TC in rats fed with a cholesterol-enriched diet. Meanwhile, serum TC and LDL-C of mice fed with a high fat (HF) diet supplemented with isorhamnetin were significantly lower than those of mice fed with HF diet alone [43]; this suggests that isorhamnetin can improve lipid metabolism disorders. *In vitro* experiments have demonstrated that taxifolin inhibits cholesterol synthesis in a dose- and time-dependent manner. Mechanistic studies have suggested that taxifolin inhibits the activity of HMG-CoA. Further, cellular cholesterol esterification and synthesis of triacylglycerol and phospholipids were also significantly suppressed in the presence of taxifolin [44]. Meanwhile, taxifolin has obvious anti-oxidant reducing ability, as well as radical scavenging and metal-chelating activity [45], which prevent lipid peroxidation and thus protect the cardiovascular system. In a word, these studies support the efficacy and diversity of DO in treating hyperlipidemia and the diversity of active ingredients.

### 5.2. Potential Targets of DO as a Treatment for Hyperlipidemia

After exploring the PPI network of DO for hyperlipidemia, the key targets of DO for hyperlipidemia included AKT1, TNF, PPARG, ADIPOQ, and APOB. These targets are primarily involved in regulating cholesterol homeostasis, cholesterol metabolic processes, and clearance of low-density lipoprotein particles.

AKT is a serine/threonine protein kinase that has been implicated in numerous cellular processes [46]. AKT1 has been linked to cholesterol-sensitive signaling mechanisms [47], and research has suggested that Akt acts on its downstream target, mTORC1 (important for autophagy), to negatively regulate cholesterol efflux to apoA-1 and thus improve circulating cholesterol levels [48]. Juan Zhong et al. [49] found that activating the AMPK/Akt/mTOR signaling pathway can ameliorate hyperlipidemia and liver steatosis. In addition, the PI3K/Akt/mTOR signaling pathway controls lipid metabolism by regulating fatty acid synthesis and the transcription factor FoxO1 [50].

As an inflammatory element, TNF is generated by activating monocytes or macrophages. While oxidizing low-density lipoprotein (ox-LDL), TNF can grow the binding of ox-LDL to endothelial cells and further improve the expression of ox-LDL receptor [51]. Existing studies have reported that TNF-*α* inhibits cholesterol efflux by causing overexpression of micro-RNA-101and inhibiting the expression of ATP-binding cassette transporter A1 (ABCA1) [52].

PPARG is a significant regulator of lipid homeostasis. Activating or inhibiting PPARG expression causes changes in the activity of proadipogenic and antiadipogenic regulators. Lipid mobilization is controlled by these processes to adipocytes by boosting adipogenesis and controlling the expression of adipocyte-secreted proteins and adipocytokines including leptin and adiponectin, decreasing adipotoxicity [53].

Adiponectin (also known as ADIPOQ) is a unique adipocytokine that includes growing insulin sensitivity, boosting fatty acid oxidation, stopping inflammatory reactions, and inducing endothelium-dependent, nitric oxide-mediated vasodilation [54]. Research shows that adiponectin promotes cholesterol efflux through interactions with the transmembrane receptors AdipoR1 and AdipoR2, suggesting that adiponectin may be an effective marker for atherosclerotic disease [55].

Apolipoprotein B (APOB) is a main structural protein in very low-density lipoprotein, intermediate-density lipoprotein, LDL, and lipoprotein (a) [56], which can remove the residue of lipid metabolism through low-density lipoprotein receptor (LDLR) mediated endocytosis on the surface of liver cells [57].

In summary, AKT1, TNF, PPARG, ADIPOQ, and APOB may be targets for the action of DO in the treatment of hyperlipidemia.

### 5.3. Molecular Mechanism of DO as a Treatment for Hyperlipidemia

Based on the KEGG enrichment and C-T-P network analysis, DO has been hypothesized to influence several key pathways, which are important in antihyperlipidemia. Those signaling pathways include the PPAR signaling pathway, insulin resistance, the AMPK signaling pathway, and nonalcoholic fatty liver disease (NAFLD).

The PPAR signaling is important in many diseases such as obesity, diabetes, and atherosclerosis. PPAR-*α* and PPAR-*γ* mainly regulate lipid metabolism, insulin sensitivity, bile acid (BA), and glucose homeostasis [58]. Similarly, PPAR-*β*/*δ* regulates lipid metabolism, glucose homeostasis, anti-inflammatory effects, and fatty acid oxidation, which are keys areas for the action of drugs for hyperlipidemia. Experimental data have suggested that naringenin regulates the activity of nuclear receptors PPAR (*α*, *γ*), resulting in decreased production of cholesterol and bile acid [59].

Insulin signaling is connected to Type 2 diabetes and related diseases like obesity, hyperlipidemia, and atherosclerosis that are caused by insulin resistance [60]. Published data have suggested that DO prevents insulin resistance in rats with diabetes mellitus (DM) [61], suggesting that DO may be effective as a treatment for hyperlipidemia through attenuating insulin resistance.

AMPK activation promotes the activation of signaling pathways related to fatty acid oxidation and inhibits anabolic ATP-consuming processes such as gluconeogenesis and synthesis of lipids and proteins [62]. AMPK has attracted widespread attention as a potential therapeutic target for metabolic diseases (including hyperlipidemia) [63]. Research has indicated that isorhamnetin (a bioactive compound contained in DO) affects AMPK activation during differentiation of 3T3-L1 adipocytes and reduces the accumulation of intracellular lipids and triglycerides, as well as decreasing glycerol-3-phosphate dehydrogenase (GPDH) activity [64].

The development of NAFLD is tightly tied to other clinical developments such as obesity, dyslipidemia, diabetes, and metabolic syndrome [65], associated with NAFLD extensively, such as PPAR, AMPK, PI3K-Akt, ER stress, TNF-*α*, and FAAs, and also associated with NAFLD progression. NAFLD also presents with atherosclerotic dyslipidemia, postprandial lipemia, and HDL dysfunction [66].

### 5.4. Experimental Validation of DO as a Treatment for Hyperlipidemia

Through preliminary network pharmacology analysis, we used PPI and KEGG analysis to get potential targets and key signaling pathways of DO against hyperlipidemia. The results indicated that NAFLD signaling pathway and other NAFLD-related signaling pathways are involved in the treatment of hyperlipidemia by DO. Then, we established a HFDA-induced hyperlipidemia rat model and supplied with DO to verify its antihyperlipidemic effect. The outcomes displayed that DO could significantly reduce TC, TG, and LDL-C, grow HDL-C in hyperlipidemia model rats, and improve the damage caused by liver and lipid metabolism disorder.

Dyslipidemia often co-existed with NAFLD/NASH, and the relationship between dyslipidemia and NAFLD is bidirectional. In this study, liver pathological section implied that DO can ameliorate hepatocyte ballooning, steatosis, and inflammation to stop NAFLD caused by lipid metabolism disorders. The lipid droplets in liver were stained with Oil red O, and it was found that DO reduced the lipid droplets, which showed that DO better influences serum TC and liver function; it is further verified that DO may treat hyperlipidemia by affecting NAFLD-related signaling pathways.

## 6. Conclusion

This research made network pharmacology and validation experiments to reveal the biochemistry foundation and underlying mechanisms of DO as a treatment for hyperlipidemia. There are multiple active compounds in DO that act on multiple distinct targets through a variety of biological processes and pathways to treat hyperlipidemia; meanwhile, validation experiments indicated that DO may treat hyperlipidemia by affecting NAFLD-related signaling pathways.

However, these conclusions are limited by their purely bioinformatic design and preliminary experimental verification. But these findings suggest that DO, a botanical treatment from TCM, may have a true pharmacological basis for a clinical effect. As such, we hope that these findings may motivate future network pharmacology-based investigations of other Chinese herbs that may be promising treatments for against hyperlipidemia and related disorders.

## Figures and Tables

**Table 1:** Alcohol consumption gradient scale.

Day	1∼4 (%)	5∼8 (%)	9∼12 (%)	13∼15 (%)	16∼20 (%)	21∼25 (%)	26∼30 (%)	After 30 days (%)
Alcohol volume fraction	4	8	12	16	19	21	22	22

**Table 2:** Chemical information of 11 active compounds in DO.

Mol ID	Molecule name	Structure	MW	OB (%)	DL
MOL004328	Naringenin	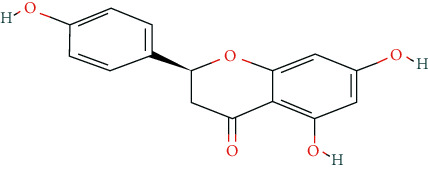	272.27	59.29	0.21
MOL002322	Isovitexin	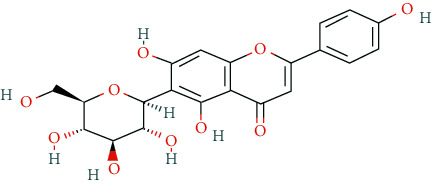	432.41	31.29	0.72
MOL005190	Eriodictyol	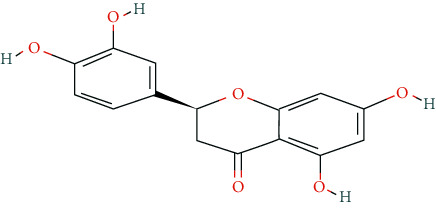	288.27	71.79	0.24
MOL003044	Chrysoeriol	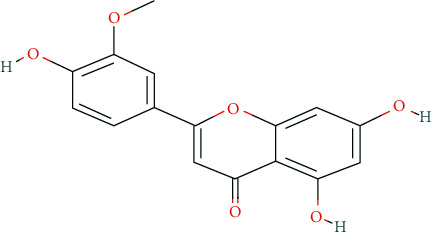	300.28	35.85	0.27
MOL001736	(-)-taxifolin	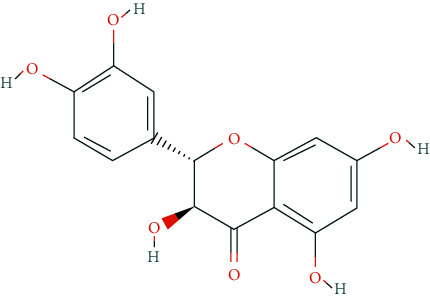	304.27	60.51	0.27
MOL004576	Taxifolin	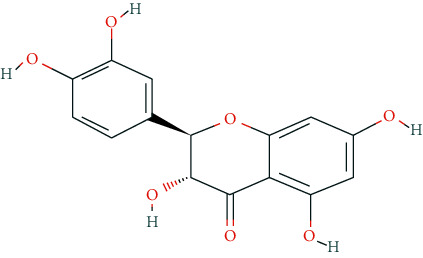	304.27	57.84	0.27
MOL000354	Isorhamnetin	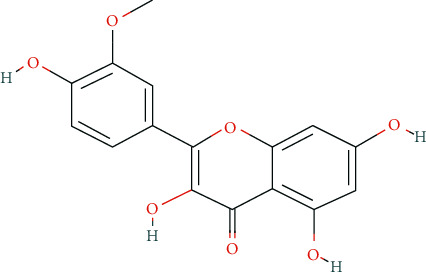	316.28	49.6	0.31
MOL000483	(Z)-3-(4-hydroxy-3-methoxy-phenyl)-N-[2-(4-hydroxyphenyl)ethyl]acrylamide	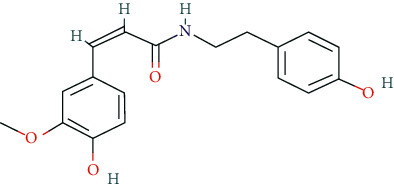	313.38	118.35	0.26
MOL000359	*β*-sitosterol	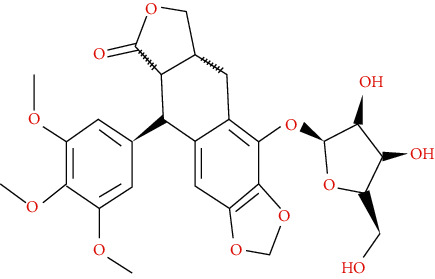	414.79	36.91	0.75
MOL003591	ar-curcumene	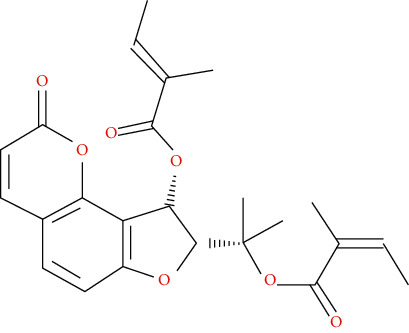	426.5	52.34	0.65
MOL008647	Moupinamide	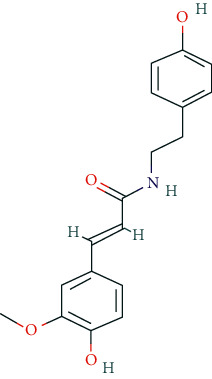	313.38	86.71	0.26

There were 264 compounds acquired from DO by various bibliographical databases, and 11 candidate molecules were selected on basis of coefficients of ADME nature (Per OB ≥ 30%, Per DL ≥ 0.18). TCMSP: The traditional Chinese medicine systems pharmacology database and analysis platform (http://tcmspw.com/index.php). ADME: Absorption (OB), distribution (DL), metabolism, and excretion. MW: molecular weight.

**Table 3:** Information of 89 candidate targets for 10 compounds. Deleted the compounds without targets on basis of the TCMSP database or had not related gene name on basis of Uniprot database. Finally, 10 compounds were acquired.

Mol ID	Protein name	Gene name
MOL004328	Prostaglandin G/H synthase 1	PTGS1
MOL004328	Estrogen receptor	ESR1
MOL004328	Prostaglandin G/H synthase 2	PTGS2
MOL004328	Heat shock protein HSP 90	HSP 90
MOL004328	Beta-lactamase	blaC
MOL004328	mRNA of PKA Catalytic Subunit C-alpha	PRKACA
MOL004328	Phosphatidylinositol-4,5-bisphosphate 3-kinase catalytic subunit, gamma isoform	PIK3CG
MOL004328	Transcription factor p65	RELA
MOL004328	RAC-alpha serine/threonine-protein kinase	AKT1
MOL004328	Apoptosis regulator Bcl-2	BCL2
MOL004328	Mitogen-activated protein kinase 3	MAPK3
MOL004328	Mitogen-activated protein kinase 1	MAPK1
MOL004328	Caspase-3	CASP3
MOL004328	Fatty acid synthase	FASN
MOL004328	Low-density lipoprotein receptor	LDLR
MOL004328	Bcl2 antagonist of cell death	BAD
MOL004328	Superoxide dismutase [Cu-Zn]	SOD1
MOL004328	Catalase	CAT
MOL004328	Peroxisome proliferator-activated receptor gamma	PPARG
MOL004328	Microsomal triglyceride transfer protein large subunit	MTTP
MOL004328	Apolipoprotein B-100	APOB
MOL004328	Phospholipase B1, membrane-associated	PLB1
MOL004328	3-hydroxy-3-methylglutaryl-coenzyme A reductase	HMGCR
MOL004328	Cytochrome P450 19A1	CYP19A1
MOL004328	Glutathione S-transferase P	GSTP1
MOL004328	UDP-glucuronosyltransferase 1-1	UGT1A1
MOL004328	Peroxisome proliferator-activated receptor alpha	PPARA
MOL004328	Sterol regulatory element-binding protein 1	SREBF1
MOL004328	Glutathione reductase, mitochondrial	GSR
MOL004328	Multidrug resistance-associated protein 1	ABCC1
MOL004328	Adiponectin	ADIPOQ
MOL004328	Sterol O-acyltransferase 2	SOAT2
MOL004328	Aldo-keto reductase family 1 member C1	AKR1C1
MOL004328	Aspartate aminotransferase, cytoplasmic	GOT1
MOL004328	4-aminobutyrate aminotransferase, mitochondrial	ABAT
MOL004328	Liver carboxylesterase 1	CES1
MOL004328	Sterol O-acyltransferase 1	SOAT1
MOL002322	Prostaglandin G/H synthase 2	PTGS2
MOL002322	Androgen receptor	AR
MOL002322	DNA topoisomerase II	TOP2
MOL002322	Transcription factor p65	RELA
MOL002322	Inhibitor of nuclear factor kappa-B kinase subunit beta	IKBKB
MOL002322	Tumor necrosis factor	TNF
MOL005190	Prostaglandin G/H synthase 1	PTGS1
MOL005190	Prostaglandin G/H synthase 2	PTGS2
MOL005190	Heat shock protein HSP 90	HSP90
MOL005190	mRNA of PKA Catalytic Subunit C-alpha	PRKACA
MOL005190	Nuclear receptor coactivator 2	NCOA2
MOL005190	Phosphatidylinositol-4,5-bisphosphate 3-kinase catalytic subunit, gamma isoform	PIK3CG
MOL005190	Heme oxygenase 1	HMOX1
MOL005190	Nuclear factor erythroid 2-related factor 2	NFE2L2
MOL005190	NAD(P)H dehydrogenase [quinone] 1	NQO1
MOL003044	Nitric oxide synthase, inducible	NOS2
MOL003044	Prostaglandin G/H synthase 1	PTGS1
MOL003044	Estrogen receptor	ESR1
MOL003044	Androgen receptor	AR
MOL003044	Peroxisome proliferator activated receptor gamma	PPARG
MOL003044	Prostaglandin G/H synthase 2	PTGS2
MOL003044	Dipeptidyl peptidase IV	DPP4
MOL003044	Mitogen-activated protein kinase 14	MAPK14
MOL003044	Glycogen synthase kinase-3 beta	GSK3B
MOL003044	Heat shock protein HSP 90	HSP90
MOL001736	Prostaglandin G/H synthase 1	PTGS1
MOL001736	Prostaglandin G/H synthase 2	PTGS2
MOL001736	Heat shock protein HSP 90	HSP90
MOL001736	Phosphatidylinositol-4,5-bisphosphate 3-kinase catalytic subunit, gamma isoform	PIK3CG
MOL004576	Prostaglandin G/H synthase 1	PTGS1
MOL004576	Prostaglandin G/H synthase 2	PTGS2
MOL004576	Heat shock protein HSP 90	HSP90
MOL004576	Phosphatidylinositol-4,5-bisphosphate 3-kinase catalytic subunit, gamma isoform	PIK3CG
MOL004576	Retinoic acid receptor RXR-alpha	RXRA
MOL004576	Aldose reductase	ALR
MOL004576	Transcription factor p65	RELA
MOL004576	Metal-binding activator 1	MAC1
MOL004576	Intercellular adhesion molecule 1	ICAM1
MOL004576	Diacylglycerol O-acyltransferase 2	DGAT2
MOL004576	Microsomal triglyceride transfer protein large subunit	MTTP
MOL004576	Apolipoprotein B-100	APOB
MOL000354	Nitric oxide synthase, inducible	NOS2
MOL000354	Prostaglandin G/H synthase 1	PTGS1
MOL000354	Estrogen receptor	ESR1
MOL000354	Androgen receptor	AR
MOL000354	Peroxisome proliferator activated receptor gamma	PPARG
MOL000354	Prostaglandin G/H synthase 2	PTGS2
MOL000354	mRNA of Protein-tyrosine phosphatase, nonreceptor type 1	PTPN1
MOL000354	Estrogen receptor beta	ESR2
MOL000354	Dipeptidyl peptidase IV	DPP4
MOL000354	Mitogen-activated protein kinase 14	MAPK14
MOL000354	Glycogen synthase kinase-3 beta	GSK3B
MOL000354	Heat shock protein HSP 90	HSP90
MOL000354	Cell division protein kinase 2	cdk-2
MOL000354	Phosphatidylinositol-4,5-bisphosphate 3-kinase catalytic subunit, gamma isoform	PIK3CG
MOL000354	mRNA of PKA Catalytic Subunit C-alpha	PRKACA
MOL000354	Trypsin-1	PRSS1
MOL000354	Proto-oncogene serine/threonine-protein kinase Pim-1	PIM1
MOL000354	Cyclin-A2	CCNA2
MOL000354	Nuclear receptor coactivator 2	NCOA2
MOL000354	Calmodulin	CALM
MOL000354	Glycogen phosphorylase, muscle form	PYGM
MOL000354	Peroxisome proliferator activated receptor delta	PPARD
MOL000354	Serine/threonine-protein kinase Chk1	CHEK1
MOL000354	Aldose reductase	ALR
MOL000354	Nuclear receptor coactivator 1	NCOA1
MOL000354	Coagulation factor VII	F7
MOL000354	Thrombin	F2
MOL000354	Nitric-oxide synthase, endothelial	NOS3
MOL000354	Acetylcholinesterase	ACHE
MOL000354	Gamma-aminobutyric acid receptor subunit alpha-1	GABRA1
MOL000354	Amine oxidase [flavin-containing] B	MAOB
MOL000354	Glutamate receptor 2	GRIA2
MOL000354	Cytochrome P450-cam	camC
MOL000354	Transcription factor p65	RELA
MOL000354	Xanthine dehydrogenase/oxidase	XDH
MOL000354	Neutrophil cytosol factor 1	NCF1
MOL000354	Oxidized low-density lipoprotein receptor 1	OLR1
MOL000483	Prostaglandin G/H synthase 1	PTGS1
MOL000483	Prostaglandin G/H synthase 2	PTGS2
MOL000483	CGMP-inhibited 3′,5′-cyclic phosphodiesterase A	PDE3A
MOL000483	Alpha-1B adrenergic receptor	ADRA1B
MOL000483	Beta-2 adrenergic receptor	ADRB2
MOL000483	Heat shock protein HSP 90	HSP90
MOL000483	Leukotriene A-4 hydrolase	LTA4H
MOL000483	Calmodulin	CALM
MOL000359	Progesterone receptor	PGR
MOL000359	Nuclear receptor coactivator 2	NCOA2
MOL000359	Mineralocorticoid receptor	NR3C2
MOL000359	Glucocorticoid receptor	NR3C1
MOL000359	mRNA of PKA Catalytic Subunit C-alpha	PRKACA
MOL000359	Heat shock protein HSP 90	HSP90
MOL000359	Aldose reductase	AKR1B1
MOL000359	Proto-oncogene serine/threonine-protein kinase Pim-1	PIM1
MOL003591	Thrombin	F2
MOL003591	Potassium voltage-gated channel subfamily H member 2	KCNH2
MOL003591	Coagulation factor Xa	F10
MOL003591	Prostaglandin G/H synthase 2	PTGS2
MOL003591	DNA topoisomerase II	TOP2A
MOL003591	Dipeptidyl peptidase IV	DPP4
MOL003591	Trypsin-1	PRSS1
MOL003591	Nuclear receptor coactivator 2	NCOA2
MOL003591	Nuclear receptor coactivator 1	NCOA1
MOL003591	Calmodulin	CAM

**Table 4:** Information of selected the top 6 Hub genes.

Gene name	Uniprot ID	Description	Protein function
AKT1	P31749	AKT serine/threonine kinase 1	Enzymes; RAS pathway related proteins.
TNF	P01375	Tumor necrosis factor	Cytokine that binds to TNFRSF1A/TNFR1 and TNFRSF1B/TNFBR, can induce cell death of certain tumor cell lines.
PPARG	P37231	Peroxisome proliferator activated receptor gamma	Nuclear receptors; transcription factors/Zinc-coordinating DNA-binding domains.
ADIPOQ	Q15848	Adiponectin	Important adipokine involved in the control of fat metabolism and insulin sensitivity, with direct antidiabetic, antiatherogenic, and anti-inflammatory activities.
APOB	P04114	Apolipoprotein B-100	APOB is a major protein constituent of chylomicrons, LDL and VLDL.APOB functions as a recognition signal for the cellular binding and internalization of LDL particles by the apoB/E receptor.
NOS3	P29474	Nitric oxide synthase, endothelial	Produces nitric oxide (NO) which is implicated in vascular smooth muscle relaxation through a cGMP-mediated signal transduction pathway.

**Table 5:** Based on the KEGG enrichment and C-T-P network analysis, we picked out 5 important signaling pathways that were significantly associated with DO treatment of hyperlipidemia.

Term	ID	Input number	*P*-value	Input gene name
PPAR signaling pathway	hsa03320	7	3.13 *E* − 06	PPARA, PPARD, OLR1, RXRA, PPARG, ADIPOQ
Insulin resistance	hsa04931	8	3.20 *E* − 06	PIK3CG, SREBF1, AKT1, PPARA, TNF, PYGM, GSK3B, NOS3
AMPK signaling pathway	hsa04152	8	8.48 *E* − 06	PIK3CG, SREBF1, AKT1, HMGCR, PPARG, FASN, ADIPOQ
Nonalcoholic fatty liver disease (NAFLD)	hsa04932	8	2.57 *E* − 05	PIK3CG, SREBF1, AKT1, PPARA, TNF, GSK3B, RXRA, ADIPOQ
Thyroid hormone signaling pathway	hsa04919	7	5.15 *E* − 05	PIK3CG, AKT1, NCOA1, NCOA2, GSK3B, RXRA, ESR1

## Data Availability

The datasets used and/or analyzed during the current study are available from Dr. Lin-Zi Li upon reasonable request.

## Abbreviations

BP: Biological processes
CC: Cellular components
C-T: Compound-target
C-T-P: Compound-targets-pathway
DL: Drug-likeness
DO: Dendrobium officinale
GO: Gene ontology
H&E: Hematoxylin-eosin
HFDA: High-sucrose-fat diet and alcohol
KEGG: Kyoto Encyclopedia of Genes and Genomes
MF: Molecular functions
OB: Oral bioavailability
PPI: Protein-protein interaction
TCM: Traditional Chinese Medicine
TCMSP: Traditional Chinese Medicine Systems Pharmacology.

## Supplementary Materials

Supplementary Materials Table S1: chemical compounds in DO. Table S2: 139 compounds with structural information in DO. Table S3: the detailed information of hyperlipidemia-related targets. Table S4: compounds in PS and their corresponding targets. Table S5: common targets. Table S6: a protein-protein interaction (PPI) network of DO treating hyperlipidemia. Table S7: information of selected the top 6 Hub genes. Table S8: GO. Table S9: KEGG analysis. Table S10: C-T-P network analysis.
